# Association Between Tumor Necrosis Factor Inhibitor Exposure and Inflammatory Central Nervous System Events

**DOI:** 10.1001/jamaneurol.2020.1162

**Published:** 2020-05-18

**Authors:** Amy Kunchok, Allen J. Aksamit, John M. Davis, Orhun H. Kantarci, B. Mark Keegan, Sean J. Pittock, Brian G. Weinshenker, Andrew McKeon

**Affiliations:** 1 Department of Neurology, Mayo Clinic, Rochester, Minnesota; 2 Division of Rheumatology, Mayo Clinic, Rochester, Minnesota; 3 Department of Laboratory Medicine and Pathology, Mayo Clinic, Rochester, Minnesota

## Abstract

**Importance:**

Tumor necrosis factor (TNF) inhibitors are common therapies for certain autoimmune diseases, such as rheumatoid arthritis. An association between TNF inhibitor exposure and inflammatory central nervous system (CNS) events has been postulated but is poorly understood.

**Objective:**

To evaluate whether TNF inhibitor exposure is associated with inflammatory demyelinating and nondemyelinating CNS events in patients with an indication for TNF inhibitor use and to describe the spectrum of those CNS events.

**Design, Setting, and Participants:**

A nested case-control study was conducted using the medical records of patients with autoimmune diseases treated at 3 Mayo Clinic locations (Rochester, Minnesota; Scottsdale, Arizona; and Jacksonville, Florida) between January 1, 2003, and February 20, 2019. Patients were included if their records reported *International Statistical Classification of Diseases and Related Health Problems, Tenth Revision,* diagnostic codes for US Food and Drug Administration–approved autoimmune disease indication for TNF inhibitor use (ie, rheumatoid arthritis, ankylosing spondylitis, psoriasis and psoriatic arthritis, Crohn disease, and ulcerative colitis) and diagnostic codes for inflammatory CNS events of interest. Patients were matched 1:1 with control participants by year of birth, type of autoimmune disease, and sex.

**Exposures:**

TNF inhibitor exposure data were derived from the medical records along with type of TNF inhibitor, cumulative duration of exposure, and time of exposure.

**Main Outcomes and Measures:**

The main outcome was either inflammatory demyelinating (multiple sclerosis and other diseases such as optic neuritis) or nondemyelinating (meningitis, meningoencephalitis, encephalitis, neurosarcoidosis, and CNS vasculitis) CNS event. Association with TNF inhibitor was evaluated with conditional logistic regression and adjusted for disease duration to determine the odds ratios (ORs) and 95% CIs. Secondary analyses included stratification of outcome by inflammatory demyelinating and nondemyelinating CNS events and by autoimmune disease (rheumatoid arthritis and non–rheumatoid arthritis).

**Results:**

A total of 212 individuals were included: 106 patients with inflammatory CNS events and 106 control participants without such events. Of this total, 136 were female (64%); the median (interquartile range) age at disease onset for patients was 52 (43-62) years. Exposure to TNF inhibitors occurred in 64 patients (60%) and 42 control participants (40%) and was associated with an increased risk of any inflammatory CNS event (adjusted OR, 3.01; 95% CI, 1.55-5.82; *P* = .001). These results were similar when the outcome was stratified by demyelinating and nondemyelinating CNS events. Secondary analyses found the association was predominantly observed in patients with rheumatoid arthritis (adjusted OR, 4.82; 95% CI, 1.62-14.36; *P* = .005).

**Conclusions and Relevance:**

This study found that exposure to TNF inhibitors in patients with autoimmune diseases appeared to be associated with increased risk for inflammatory CNS events. Whether this association represents de novo or exacerbated inflammatory pathways requires further research.

## Key Points

**Question:**

Is exposure to tumor necrosis factor inhibitors associated with risk of inflammatory demyelinating and nondemyelinating central nervous system events in patients with an autoimmune disease?

**Findings:**

In this case-control study of 212 patients with or without inflammatory CNS events, exposure to tumor necrosis factor inhibitors was associated with an increased risk of inflammatory central nervous system events. The association was similar for both inflammatory demyelinating and nondemyelinating central nervous system events.

**Meaning:**

The association observed between exposure to tumor necrosis factor inhibitor and increased risk of inflammatory demyelinating and nondemyelinating central nervous system events warrants future research to ascertain whether the association may indicate de novo inflammation or exacerbation of already aberrant inflammatory pathways.

## Teaser

This case-control study examines the inflammatory CNS event outcome among patients with an autoimmune disease who were treated with tumor necrosis factor inhibitors.

## Introduction

Autoimmune diseases such as inflammatory bowel disease (eg, Crohn disease, ulcerative colitis) and autoimmune rheumatic diseases such as rheumatoid arthritis, ankylosing spondylitis, psoriasis, and psoriatic arthritis are commonly treated with highly effective immunotherapies such as tumor necrosis factor (TNF) inhibitors.^1,2,3^ Five TNF inhibitors (etanercept, infliximab, adalimumab, golimumab, and certolizumab pegol) have been approved by the US Food and Drug Administration (FDA) for these diseases.

After the introduction of TNF inhibitors, several studies reported an association between TNF inhibitors and inflammatory demyelinating central nervous system (CNS) events such as optic neuritis, transverse myelitis and multiple sclerosis (MS), and neuromyelitis optica spectrum disorder (NMOSD). Two patients with MS had an increase in gadolinium-enhancing brain lesions and inflammatory cerebrospinal fluid during treatment with infliximab.^4^ Subsequently, a randomized clinical trial of lenercept for MS was terminated because of the increased attack frequency and severity in individuals receiving active treatment.^5^ Numerous case reports over the past 20 years have included adverse events reported to the FDA: 740 inflammatory demyelinating CNS events (of which 254 were MS) collected in the BIOGEAS Registry (a Spanish multicenter study) and 358 optic neuritis cases (included in the BIOGEAS data) analyzed in the Casey Eye Institute study.^6,7,8^

Little is known about the inflammatory nondemyelinating CNS events that occur during TNF inhibitor therapy. Case reports have suggested a possible association between nondemyelinating CNS events and neurosarcoidosis, CNS vasculitis, leptomeningitis, or meningoencephalitis.^9,10,11,12,13,14^

Although the association between TNF inhibitor exposure and inflammatory CNS events has been postulated from previous studies, inflammatory CNS events can occur without TNF inhibitor exposure, and reports exist predating the TNF inhibitor treatment era.^15,16^ Furthermore, inflammatory demyelinating CNS events may occur with a higher frequency in patients with autoimmune diseases as demonstrated by familial clusters and case-control disease studies.^17,18,19^

In this nested case-control study, we evaluated patients with autoimmune diseases with an FDA–approved indication for TNF inhibitor use. The goal was to ascertain whether TNF inhibitor treatment was associated with an increased risk for inflammatory demyelinating and nondemyelinating CNS events.

## Methods

### Study Design and Study Population

This nested case-control study included patients selected from a population at the Mayo Clinic, a tertiary referral clinical practice. We used the Advanced Cohort Explorer, the searchable electronic medical record system of the Mayo Clinic, to identify patients with autoimmune diseases treated at the clinic’s Rochester, Minnesota; Scottsdale, Arizona; and Jacksonville, Florida, locations between January 1, 2003, and February 20, 2019. Patients included were those with *International Statistical Classification of Diseases and Related Health Problems, Tenth Revision,* (*ICD-10*) diagnostic codes restricted to the following FDA–approved indications for TNF inhibitor use: rheumatoid arthritis, ankylosing spondylitis, psoriasis and psoriatic arthritis, Crohn disease, and ulcerative colitis (eFigure 1 in the Supplement). This study was approved by the institutional review board of the Mayo Clinic. All patients provided written informed consent for participation in this study.

To maximize the reliability of the clinical diagnosis, we selected patients whose medical record included use of a medication from a list of disease-modifying therapies (DMT) for autoimmune diseases (eTable 1 in the Supplement). The technique of combining a clinical diagnosis with DMT exposure has been demonstrated in data registry studies to increase the positive predictive value of a confirmed diagnosis of rheumatoid arthritis.^20^

### Selection of Patients With CNS Inflammation

Patients included in the study had either inflammatory demyelinating or nondemyelinating CNS events. All of these patients were first identified with *ICD-10* diagnostic codes and then confirmed with medical record review (eFigure 1 in the Supplement). The date of the inflammatory CNS event symptom onset was assigned as the index date.

Inflammatory demyelinating CNS events required a neurologist’s diagnosis of relapsing-remitting MS,^21^ primary progressive MS,^21^ clinically isolated syndrome,^21^ radiologically isolated syndrome,^21^ NMOSD,^22^ and transverse myelitis. Optic neuropathy was a clinical diagnosis made by either a neurologist or an ophthalmologist with supportive ancillary tests for visual acuity, color and visual field, visual evoked potentials, and inflammatory changes of the optic nerve on magnetic resonance imaging or ocular coherence tomography. Patients with optic neuropathies attributed to noninflammatory causes such as trauma, ischemia, or medication were excluded (eTable 2 in the Supplement).

Inflammatory nondemyelinating CNS events included meningitis, meningoencephalitis, encephalitis, neurosarcoidosis, and CNS vasculitis, without documented alternative causes (eg, infection, neoplasia) (eTable 2 in the Supplement). Each clinical diagnosis was accompanied by supportive ancillary testing; cerebrospinal fluid with leukocytosis (>5 white cells per high-powered field); or magnetic resonance imaging scan of the brain or spine demonstrating leptomeningeal, pachymeningeal, parenchymal, or vascular pathology.

### Selection of Control Participants Without CNS Inflammation

The R software package MatchIt, version 3.0.2 (R Foundation for Statistical Computing), was used to match control participants 1:1, with exact match for sex, year of birth, and type of autoimmune disease (rheumatoid arthritis, psoriasis and psoriatic arthritis, ankylosing spondylitis, Crohn disease, and ulcerative colitis) (eFigure 1 in the Supplement). Review of the medical records confirmed that the control participants had the matched autoimmune rheumatic disease or inflammatory bowel disease and did not have inflammatory CNS events. All control participants were assigned the index date of their matched patients. Each control participant was required to have had an autoimmune disease onset and to be alive before their index date. Control participants were required to have follow-up within 6 months of the matched index date, to ensure they were followed up until approximately the same age (eFigure 2 in the Supplement).

### Exclusions

Any inflammatory CNS events that developed before the autoimmune disease diagnosis were excluded. Any patients with CNS vasculitis or CNS sarcoidosis who had preexisting systemic vasculitis or systemic sarcoidosis were excluded (eTable 2 of the Supplement). Individuals with preexisting immunodeficiency or an immunocompromised system in either cohort were excluded because this condition may preclude TNF inhibitor therapy or may predispose these individuals to developing inflammatory CNS events (eTable 2 of the Supplement). No patient could contribute as 2 cases.

### Exposures

All medical records of patients and control participants were reviewed to identify their exposure to TNF inhibitors. The type of TNF inhibitor, cumulative duration of exposure, and time of exposure in relation to the index date (for patients) were derived from the medical records.

### Statistical Analysis

To account for the potential association of age, sex, or type of autoimmune disease with the development of an inflammatory CNS event, we performed a matched analysis. A conditional logistic regression model was created to identify the odds ratios (ORs) and 2-sided 95% CIs for the association of inflammatory CNS events with any TNF inhibitor exposure. The model was analyzed for the effect of the binary variable of TNF inhibitor exposure (before the index date), and the model was adjusted for disease duration (disease onset until the index date). Statistical power required for the model was estimated by calculating a minimum of 10 events per variable.^23^ All evaluations were conducted from August 2018 to August 2019, using R software, version 3.5.1 (R Foundation for Statistical Computing).

## Results

### Study Cohort and Baseline Characteristics

The study population included 32 043 patients in 3 Mayo Clinic locations with *ICD-10* diagnostic codes for rheumatoid arthritis, psoriasis and psoriatic arthritis, ankylosing spondylitis, Crohn disease, and ulcerative colitis who were ever treated with a DMT. Of this number, 929 patients had *ICD-10* diagnostic codes for inflammatory CNS events of interest; 823 were excluded because of the lack of neurological diagnosis, an alternative neurological diagnosis, disease occurring outside the study period, or an immunocompromised system. A total of 212 individuals (136 women [64%]) were analyzed, including 106 patients and 106 control participants. The median (interquartile range [IQR]) age at disease onset for patients was 52 (43-62) years.

Women represented 68 (64%) individuals in the patient group; 40 (71%) had inflammatory demyelinating CNS events, and 28 (56%) had inflammatory nondemyelinating CNS events (Table 1). Median (IQR) age at autoimmune disease onset was 36 (25-48) years for the patients and 35 (23-46) years for the control participants. Median (IQR) disease duration at the index date was 12 (5-19) years for the patients and 13 (6-22) years for the control participants. Rheumatoid arthritis was the most common autoimmune disease in the patient group (n = 48 [45%]) (Table 1). The underlying autoimmune diseases stratified by the inflammatory demyelinating and nondemyelinating CNS events are presented in eTable 3 in the Supplement.

**Table 1.** Baseline Characteristics of Patients and Control Participants

Variable	Primary cohort		Stratification			
			Inflammatory demyelinating CNS events		Inflammatory nondemyelinating CNS events	
	Control participants	Patients	Control participants	Patients	Control participants	Patients
Patients, No.	106	106	56	56	50	50
Women, No. (%)	68 (64)	68 (64)	40 (71)	40 (71)	28 (56)	28 (56)
Age at autoimmune disease diagnosis, median (IQR), y	35 (23-46)	36 (25-48)	34 (20-41)	34 (24-43)	36 (26-53)	42 (26-59)
Autoimmune disease, No. (%)						
Rheumatoid arthritis	48 (45)	48 (45)	26 (46)	26 (46)	22 (44)	22 (44)
Ankylosing spondylitis	4 (4)	4 (4)	1 (2)	1 (2)	3 (6)	3 (6)
Psoriasis and psoriatic arthritis	21 (20)	21 (20)	12 (21)	12 (21)	9 (18)	9 (18)
Crohn disease	27 (25)	27 (25)	14 (25)	14 (25)	13 (26)	13 (26)
Ulcerative colitis	6 (6)	6 (6)	3 (5)	3 (5)	3 (6)	3 (6)
Disease duration at index date, median (IQR), y	13 (6-22)	12 (5-19)	13 (6-19)	12 (6-19)	15 (8-25)	8 (4-17)

Abbreviations: CNS, central nervous system; IQR, interquartile range.

### Cases

A total of 56 patients with inflammatory demyelinating CNS events were identified: 48 (86%) had MS, optic neuritis, NMOSD, or transverse myelitis, and 8 (14%) had optic neuritis. Most patients had relapsing-remitting MS (20 [36%]) or clinically isolated syndrome (17 [30%]) (Figure, A; Table 2). Four patients (7%) had primary progressive MS, 3 (5%) had radiologically isolated syndrome, 3 (5%) had aquaporin-4-IgG–positive NMOSD, and 1 (2%) had myelin oligodendrocyte glycoprotein-IgG–associated transverse myelitis. Among the 8 patients with idiopathic optic neuritis, the condition was unilateral in 6 (11%) and bilateral in 2 (4%), with aquaporin-4-IgG–positive NMOSD in 1 (Table 2).

**Figure.**
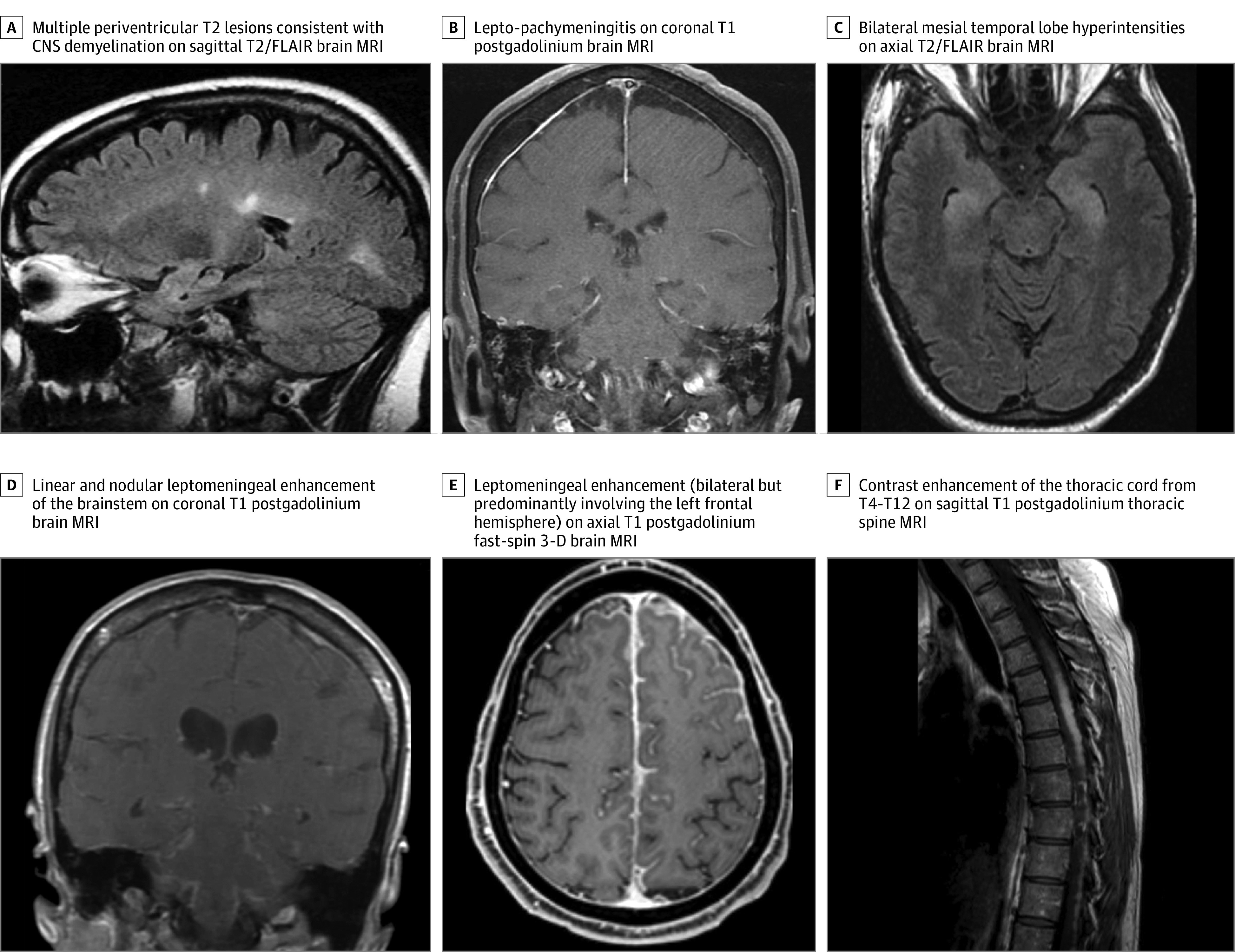
Magnetic Resonance Imaging (MRI) Features of Some Patients A, A patient with Crohn disease for 3 years who was treated with adalimumab for 10 years presented with a thoracic sensory band and lower-limb paresthesia. The patient had a cervical spine MRI that revealed multiple short-segment T2 hyperintense lesions with contrast enhancement. The diagnosis was central nervous system (CNS) demyelination. B, A patient with a history of rheumatoid arthritis who was treated with adalimumab for 16 years presented with headaches and a generalized tonic-clonic seizure; an infectious and malignant neoplasm evaluation of cerebrospinal fluid (CSF) had a negative result. Dural biopsy results revealed necrotizing meningitis, and were negative for special stains for microorganisms, including mycobacteria and fungi. The diagnosis was idiopathic lepto-pachymeningitis. C, A patient with ankylosing spondylitis and type 1 diabetes was treated with several tumor necrosis factor (TNF) inhibitors for a cumulative total of 2 years (infliximab twice, etanercept, and adalimumab). The patient had ceased using TNF inhibitor 10 months before presenting with acute confusion. The CSF analysis and positron emission tomography (PET) scan had a negative result for neural antibodies and malignant neoplasm. Serum autoimmune neural antibodies had a positive result (serum acetylcholine receptor modulating antibody, 34% loss; GAD65 antibody, 0.35 nmol/L; striational antibody, 1:240). The diagnosis was autoimmune encephalitis. The patient had a clinically robust response to intravenous methylprednisolone acetate. D, A patient with Crohn disease for 10 years was treated with adalimumab for 6 months, then infliximab for 6 months before presenting with new-onset headaches, hearing loss, paresthesias, left facial droop, numbness and diplopia. In addition to a brain MRI scan showing leptomeningeal enhancement of the brainstem, an MRI of the cervical and thoracic spine also revealed patchy leptomeningeal enhancement of the spinal cord. Results of an infectious and neoplastic evaluation of the CSF were negative. A PET scan identified increased fluorodeoxyglucose (FDG) uptake in the mediastinum, and a biopsy of subcarinal lymph node confirmed granulomatous disease. Special stains for microorganisms, including mycobacteria and fungi, had a negative result. The diagnosis was neurosarcoidosis. E, A patient with rheumatoid arthritis for 8 years who was treated with etanercept and methotrexate sodium presented with new-onset headaches and paresthesia of the right face and limbs. A right frontal biopsy specimen demonstrated necrotizing granulomatous inflammation that extensively involved the leptomeninges. Special stains for microorganisms, including mycobacteria and fungi, had a negative result. The diagnosis was neurosarcoidosis. F, A patient with a history of Crohn disease who was treated with infliximab for 3 years presented with myelopathic symptoms. A PET scan showed moderately intense FDG activity in the thoracic spinal cord. A thoracic cord biopsy demonstrated necrotizing granulomatous inflammation. Special stains for microorganisms, including mycobacteria and fungi, had a negative result. The diagnosis was neurosarcoidosis. FLAIR indicates fluid-attenuated inversion recovery sequence.

**Table 2.** Clinical, Radiological, and Serological Characteristics of Patients

Characteristic	No.	Autoimmune disease, No.	Inflammatory CNS event, No.	Median (range), y		Autoantibody, No.	Neuroimaging, No./total	CSF OCB, No./total	CSF pleocytosis, No./total	Biopsy, No.	TNF inhibitor exposure, No. (%)
				Age at inflammatory CNS event onset	Disease duration at inflammatory CNS event onset						
Demyelinating CNS events											
Demyelination	48	RA = 24; CD = 11; P/PA = 9; UC = 3; AS = 2	RRMS = 20; CIS = 17; PPMS = 4; RIS = 3; AQP4-IgG NMOSD = 3; MOG-IgG TM = 1		12 (6-18)	AQP4-IgG NMOSD = 3; MOG-IgG = 1	MRI, features of demyelination of brain or spinal cord = 48/48	20/41	10/41	NA	33 (69)
Optic neuritis	8	CD = 4; RA = 2; P/PA = 3	UON = 6; BON = 2; (AQP4-IgG NMOSD = 1)	60 (53-62)	15 (8-27)	AQP4-IgG NMOSD = 1	MRI = 3/8 with UON; 0/8 with demyelination of brain	0/3	0/3	NA	6 (75)
Nondemyelinating CNS events											
Meningitis	23	RA = 13; P/PA = 5; CD = 3; UC = 2	Aseptic (CSF only) = 8; leptomeningitis = 9; pachymeningitis = 6	68 (47-78)	10 (4-29)	NA	MRI, leptomeningitis = 9/23; pachymeningitis = 6/23 (all aseptic had negative result on MRI)	1/13	18/23 (All aseptic had pleocytosis)	Leptomeningeal = 3 (leptomeningeal inflammation); pachymeningeal = 2 (inflammation of the dura)	Aseptic = 3 (38); lepto-pachymeningitis = 9 (60)
Meningoencephalitis	4	RA = 2; P/PA = 1; AS = 1	Meningoencephalitis = 4; cerebellitis with meningeal = 1	61 (56-65)	4 (2-8)	GFAP-IgG = 2	MRI, abnormal leptomeningitis and parenchyma = 4/4	3/3	4/4	Brain (temporal, cerebellar, frontal) = 3 (leptomeningeal and parenchymal acute and chronic inflammation)	3 (75)
Encephalitis	7	CD = 4; AS = 2; RA = 1	Limbic = 3; brainstem = 1; frontal lobe = 1; multifocal = 1; cerebellitis = 1	45 (42-46)	9 (5-19)	Limbic encephalitis = 2 (patient 1: serum GAD65 = 0.09; patient 2: serum acetylcholine receptor modulating antibody = 34% loss; GAD65 antibody = 0.35 nmol/L; striational antibody = 1:240)	MRI, abnormal brain parenchyma = 7/7	1/5	6/7	Brain (frontal) = 1 (microglial activation and chronic inflammation)	3 (43)
Neurosarcoidosis	8	RA = 4; CD = 3; AS = 1	Leptomeningeal = 3; pachymeningeal = 3; cavernous sinus = 1; myelopathy = 1	59 (54-64)	16 (13-16)	NA	MRI, abnormal brain or spinal cord = 8/8	1/6	6/8	Brain = 4; pulmonary = 1; lymph node = 2; spinal cord = 1 (granulomatous disease)	7 (88)
CNS vasculitis	8	CD = 3; RA = 2; P/PA = 2; UC = 1	CNS vasculitis = 8	51 (44-65)	5 (3-9)	NA	MRI, radiological features of vasculitis = 6/8; DSA = 6/7 features of vasculitis	0/5	6/7	Brain = 2 (perivascular chronic inflammation, hemorrhage and small vein microthrombi	0

Abbreviations: AQP4-IgG NMOSD, aquaporin-4-IgG–positive neuromyelitis optica spectrum disorder; AS, ankylosing spondylitis; BON, bilateral optic neuritis; CD, Crohn disease; CIS, clinically isolated syndrome; CNS, central nervous system; CSF, cerebrospinal fluid; DSA, digital subtraction angiogram; GAD, glutamic acid decarboxylase; GFAP, glial fibrillary acidic protein; MOG-IgG, myelin oligodendrocyte glycoprotein-IgG–associated disorder; MRI, magnetic resonance imaging; NA, not applicable; OCB, oligoclonal bands; PPMS, primary progressive multiple sclerosis; P/PA, psoriasis and psoriatic arthritis; RA, rheumatoid arthritis; RIS, radiologically isolated syndrome; RRMS, relapsing-remitting multiple sclerosis; TM, transverse myelitis; UC, ulcerative colitis; UON, unilateral optic neuritis.

Of the 50 patients with inflammatory nondemyelinating CNS events, 8 had aseptic meningitis (16%), 10 had idiopathic leptomeningitis (20%), 6 had idiopathic pachymeningitis (12%; Figure, B), 5 had idiopathic meningoencephalitis (10%), 7 had autoimmune encephalitis (14%; Figure, C), 4 had neurosarcoidosis (8%; Figure, D-F), and 8 had CNS vasculitis (16%; Table 2).

### Exposure to TNF Inhibitor Therapies

Among 106 patients, 64 (60%) were exposed to TNF inhibitors compared with 42 control participants (40%). Of the individuals who had inflammatory demyelinating CNS events, 39 patients (70%) were exposed compared with 28 control participants (50%). Of the individuals who had inflammatory nondemyelinating CNS events, 25 patients (50%) were exposed compared with 14 control participants (28%). Of the patients with available data, 54 of 62 (84%) were exposed to 1 or more TNF inhibitors within 3 months of their diagnosis and 56 of 62 (90%) were exposed within 12 months. The cumulative median (IQR) duration of exposure was 2.1 (0.9-5.9) years for the patients and 3.3 (1-5.6) years for the control participants (Table 3). The TNF inhibitors to which patients were exposed are listed in Table 3.

**Table 3.** Exposure to Tumor Necrosis Factor Inhibitors Within the Study Population

Variable	No. (%)	
	Control participants	Patients
Included patients	106	106
Exposure to TNF inhibitors	42 (40)	64 (60)
Recent exposure of patients at index date		
<3 mo	NA	52 (84)
<1 y	NA	56 (90)
Missing data	NA	2 (2)
Cumulative duration of TNF inhibitor exposure at index date, median (IQR), y	3.3 (1.0-5.6)	2.1 (0.9-5.9)
Missing data	1 (1)	9 (9)
TNF inhibitors used		
Adalimumab	21 (20)	35 (33)
Infliximab	20 (19)	28 (26)
Etanercept	16 (15)	29 (27)
Certrolizumab	1 (1)	3 (3)
Gomalimumab	1 (1)	0

Abbreviations: IQR, interquartile range; NA, not applicable; TNF, tumor necrosis factor.

Of the individuals who had inflammatory demyelinating CNS events, 33 of 48 patients (69%) and 6 of 8 patients with optic neuritis (75%) were exposed to TNF inhibitors (Table 2). Of those who had inflammatory nondemyelinating CNS events, exposure to TNF inhibitors occurred in 7 of 8 patients with neurosarcoidosis (88%), 3 of 4 patients with meningoencephalitis (75%), 9 of 15 patients with lepto-pachymeningitis (60%), 3 of 7 patients with encephalitis (43%), and 3 of 8 patients with aseptic meningitis (38%) (Table 2). None of the patients with CNS vasculitis were exposed before disease onset. Of the patients exposed, the median (range) number of TNF inhibitors to which they were exposed was 2 (1-3) for patients and 2 (1-4) for control participants.

In a conditional logistic regression model adjusted for disease duration, exposure to TNF inhibitors was associated with an increased risk of developing any inflammatory CNS events (adjusted OR, 3.01; 95% CI, 1.55-5.82; *P* = .001) in patients with all autoimmune diseases.

### Secondary Analyses 

Stratification of the outcome by inflammatory demyelinating and nondemyelinating CNS events demonstrated a similar risk (adjusted OR, 3.09; 95% CI, 1.19-8.04; *P* = .02) for inflammatory demyelinating CNS events and for inflammatory nondemyelinating CNS events (adjusted OR, 2.97; 95% CI, 1.15-7.65; *P* = .02) (Table 4).

**Table 4.** Association of Inflammatory CNS Events With Exposure to Tumor Necrosis Factor Inhibitors

Variable	Outcome	Patients and control participants, No.	No. (%)		OR (95% CI)	*P* value
			Exposed patients	Exposed control participants		
Primary analysis						
All autoimmune diseases		212	64 (60)	42 (40)	3.01 (1.55-5.82)	.001
Secondary analyses stratified by autoimmune disease						
RA	Any inflammatory CNS event	96	30 (63)	16 (33)	4.82 (1.62-14.36)	.005
Non-RA (AS, P/PA, CD, UC)	Any inflammatory CNS event	116	34 (59)	26 (45)	2.13 (0.90-5.05)	.09
Secondary analyses stratified by outcome						
All autoimmune diseases	Inflammatory demyelinating CNS events	112	39 (70)	28 (50)	3.09 (1.19-8.04)	.02
All autoimmune diseases	Inflammatory nondemyelinating CNS events	100	25 (50)	14 (28)	2.97 (1.15-7.65)	.02

Abbreviations: AS, ankylosing spondylitis; CD, Crohn disease; CNS, central nervous system; OR, odds ratio; P/PA, psoriasis and psoriatic arthritis; RA, rheumatoid arthritis; UC, ulcerative colitis.

In the rheumatoid arthritis subgroup (n = 96), exposure to TNF inhibitors was associated with greater risk of developing any inflammatory demyelinating or nondemyelinating CNS events (adjusted OR, 4.82; 95% CI, 1.62-14.36; *P* = .005) (Table 4). In a pooled cohort of patients with ankylosing spondylitis, psoriasis and psoriatic arthritis, Crohn disease, and ulcerative colitis (n = 116), exposure to TNF inhibitors was not associated with a risk of developing inflammatory CNS diseases (adjusted OR, 2.13; 95% CI, 0.90-5.05; *P* = .09). Because of the lack of power, further stratification by individual autoimmune diseases was not analyzed.

## Discussion

The association between inflammatory demyelinating CNS events and TNF inhibitor exposure has long been postulated, and findings from this case-control study supported this association. Furthermore, this study demonstrated that the breadth of this association included inflammatory nondemyelinating CNS events, with a similar risk of exposure to TNF inhibitors. Secondary analyses suggested this association was predominantly seen in patients with rheumatoid arthritis.

Despite previous reports of inflammatory demyelinating CNS events, including early studies of patients with MS treated with infliximab and lenercept and numerous reports collated in the BIOGEAS Registry, the role of TNF inhibitor exposure in inflammatory demyelinating CNS events has been debated.^4,5,6,7,8^ Against an association of demyelination with TNF inhibitors are clusters of inflammatory bowel disease and autoimmune rheumatic disease in families with MS and case-control studies of inflammatory bowel disease that identified a higher frequency of MS among patients compared with control participants, which suggest a predisposition for developing inflammatory CNS events.^17,18,19^ A study of data from the Danish rheumatologic registry linked with data from the Danish MS registry reported an increased risk of MS in male patients with rheumatoid arthritis and ankylosing spondylitis who were treated with TNF inhibitors, but the study found no overall increased rate of MS in patients with TNF inhibitor exposure.^24^ However, the authors acknowledged a limitation in using the MS data registry because cases may be classified as adverse events and not as MS, resulting in underreporting.^24^ The SABER study, which used 4 large US databases, identified 61 433 cases of optic neuritis in patients with autoimmune diseases and found no difference in the association of crude optic neuritis rates with TNF inhibitors, compared with nonbiological disease-modifying antirheumatic drugs.^7^ However, the authors were limited by their inability to review the medical records to verify the cases of optic neuritis.

The present study used a nested case-control cohort matched by type of autoimmune disease, sex, and age and found that TNF inhibitor exposure appeared to be associated with these inflammatory CNS events (both demyelinating and nondemyelinating), beyond these baseline clinical characteristics. Similar to previous studies describing a temporal association, of those individuals with inflammatory CNS events who were exposed to TNF inhibitors, exposure occurred within 12 months of neurological symptom onset in 90%.^6^

Literature on the association between TNF inhibitors and inflammatory nondemyelinating CNS events is lacking. We found that TNF inhibitor exposure occurred most often in patients with neurosarcoidosis, meningoencephalitis, and lepto-pachymeningitis (>50% exposed) (Table 2). Similar to our findings, results from several case reports and cohort studies have described meningitis or meningoencephalitis in patients with autoimmune disease (rheumatoid arthritis, ankylosing spondylitis, psoriasis and psoriatic arthritis, Crohn disease) treated with a TNF inhibitor.^25,26,27,28,29,30,31,32,33^

Tumor necrosis factor inhibitors are reported to have efficacy in treating pulmonary and CNS sarcoidosis as well as other inflammatory disorders such as Behçet disease.^34,35,36^ We identified 8 patients with de novo neurosarcoidosis, of which 7 had previous TNF inhibitor exposure. These findings corroborate those in the literature that found that neurosarcoidosis developed during TNF inhibitor treatment.^37,38,39,40^ Furthermore, 111 cases of systemic sarcoidosis were reported by the BIOGEAS Registry.^8^ This paradoxical association with sarcoidosis is not yet well understood. Adalimumab and infliximab have been shown to increase interferon γ, which is a driver of granuloma formation.^41^

We hypothesized that TNF inhibitors may further dysregulate already aberrant immune responses, triggering inflammatory CNS events in patients with certain autoimmune diseases. Tumor necrosis factor is a cytokine with a range of functions, including induction of fever, defense against pathogens, inflammation, immune regulation, inhibition of tumor growth, stimulation of vascular endothelium, and proliferation of immune cells.^42^ Proposed mechanisms for the paradoxical development of inflammatory CNS events in association with TNF inhibitor exposure include immune dysregulation from the inhibition of apoptosis of autoreactive T cells, which may then enter the CNS and cause demyelination.^43^ TNF inhibition may result in upregulation of TNF expression. Because TNF inhibitors cannot cross the blood brain barrier, a paradoxical increase in TNF within the CNS may occur.^43^ Induction of autoimmunity also has been proposed by inhibiting the TNF promotion of T-regulatory cell survival and proliferation.^44^ In this study, we were unable to ascertain whether this association indicates de novo inflammation or exacerbation of already aberrant inflammatory pathways in patients with these autoimmune diseases.

In the secondary analyses stratified by autoimmune disease, the association of inflammatory CNS events with TNF inhibitor exposure was observed in patients with rheumatoid arthritis. No association was observed in the remaining pooled autoimmune diseases, suggesting differences in the association depending on the underlying autoimmune disease. A retrospective cohort study that used different methods evaluated TNF inhibitor exposure in a large cohort of patients with inflammatory bowel disease; it did not find a difference in the risk of developing an inflammatory demyelinating disease in patients exposed to TNF inhibitors.^45^ Because of the rarity of these inflammatory CNS events, we were limited by power to analyze each autoimmune disease and its association with TNF inhibitor exposure.

### Strengths and Limitations

This study has some strengths. First, it has a nested case-control study design that matched each patient with a control participant from the same population, reducing the likelihood of selection and referral bias. The patient group had both a diagnosis and a history of any DMT treatment, increasing the specificity of the autoimmune disease diagnosis.^20^ Second, beyond the *ICD-10* diagnostic codes, the study used medical records to confirm the clinical diagnoses of the underlying autoimmune disease and the inflammatory CNS event outcome, increasing the specificity of the findings. This confirmation ensured that no cases secondary to causes such as malignant neoplasms, drugs, or infections were included. Third, by matching the year of birth and the index date between patients and control participants, we accounted for the role of age in the disease course. The control participants had a median disease duration longer than that for the patients because of a positive selection for control participants who had follow-up time until the matched index date (control participants were excluded if they had follow-up time that was less than that for their matched patients). This approach strengthened the study as it ensured that control participants had adequate follow-up time to develop an inflammatory CNS event. This study has several limitations, including small samples sizes and referral bias to the Mayo Clinic (tertiary referral center), that prevent its generalizability to primary or private community practices. Potential confounding was likely because of indication bias, with TNF inhibitors being administered to patients with moderate to severe disease.^46,47^ However, we selected the control participants from the same study population as the patients, which required the patients with autoimmune disease to have been treated at some point with a DMT. This approach reduced the likelihood of matched control participants not having disease activity during the course of their autoimmune disease. Furthermore, we adjusted for disease duration, which has a clinical association with disease severity. Disease duration is also clinically associated with the introduction of second-line therapy.

Tumor necrosis factor inhibitors remain highly effective therapies for autoimmune diseases, and these associated inflammatory CNS events likely represent uncommon events. This study does not imply causality and is not a comparative study of the individual TNF inhibitor but rather the association between inflammatory CNS events and exposure to TNF inhibitors. Conclusions should not be drawn as to the associations with specific types of TNF inhibitor given that the TNF inhibitors examined in this cohort likely depict individual and institutional prescribing practices, duration of time on the market, and patient preferences and insurance policies. Although we are not cautioning against the use of these therapies, we recommend further investigation, including population-based studies, to identify the observed incidence of inflammatory CNS events and to develop multi-institutional prospectively collected registry data.

## Conclusions

In this study population, TNF inhibitor exposure in patients with autoimmune diseases appeared to be associated with an increased risk of both inflammatory demyelinating and nondemyelinating CNS events. Further research is needed to explore whether this association indicates de novo inflammation or exacerbation of already aberrant inflammatory pathways.

## Notes

Supplement. eFigure 1. Case-Control Inclusion Criteria eFigure 2. Study Design eTable 1. Disease Modifying Therapies Used by the Study Population at Any Point in Their Disease Course eTable 2. Exclusions from CNS Diagnostic Code Search for Cases eTable 3. Cases Stratified by Inflammatory Demyelinating and Non-demyelinating CNS Disorders for Each Systemic Autoimmune Disease
