# Supplementary material for: Association Between Tumor Necrosis Factor Inhibitor Exposure and Inflammatory Central Nervous System Events
Source: JAMA Neurol. 2020 May 18;77(8):1–11. doi: 10.1001/jamaneurol.2020.1162 (PMC7235930; doi:10.1001/jamaneurol.2020.1162)
Supplement: Supplement. — eFigure 1. Case-Control Inclusion Criteria eFigure 2. Study Design eTable 1. Disease Modifying Therapies Used by the Study Population at Any Point in Their Disease Course eTable 2. Exclusions from CNS Diagnostic Code Search for Cases eTable 3. Cases Stratified by Inflammatory Demyelinating and Non-demyelinating CNS Disorders for Each Systemic Autoimmune Disease [file jamaneurol-77-937-s001.pdf]

# Supplementary Online Content

Kunchok A, Aksamit AJ Jr, Davis JM III, et al. Association between tumor necrosis factor inhibitor exposure and inflammatory central nervous system events. *JAMA Neurol.* Published online May 18, 2020. doi:10.1001/jamaneurol.2020.1162

**eFigure 1.** Case-Control Inclusion Criteria

**eFigure 2.** Study Design

**eTable 1.** Disease Modifying Therapies Used by the Study Population at Any Point in Their Disease Course

**eTable 2.** Exclusions from CNS Diagnostic Code Search for Cases

**eTable 3.** Cases Stratified by Inflammatory Demyelinating and Non-demyelinating CNS Events for Each Systemic Autoimmune Disease

This supplementary material has been provided by the authors to give readers additional information about their work.

© 2020 Kunchok A et al. *JAMA Neurology.*

**eFigure 1: Case-Control Inclusion Criteria**

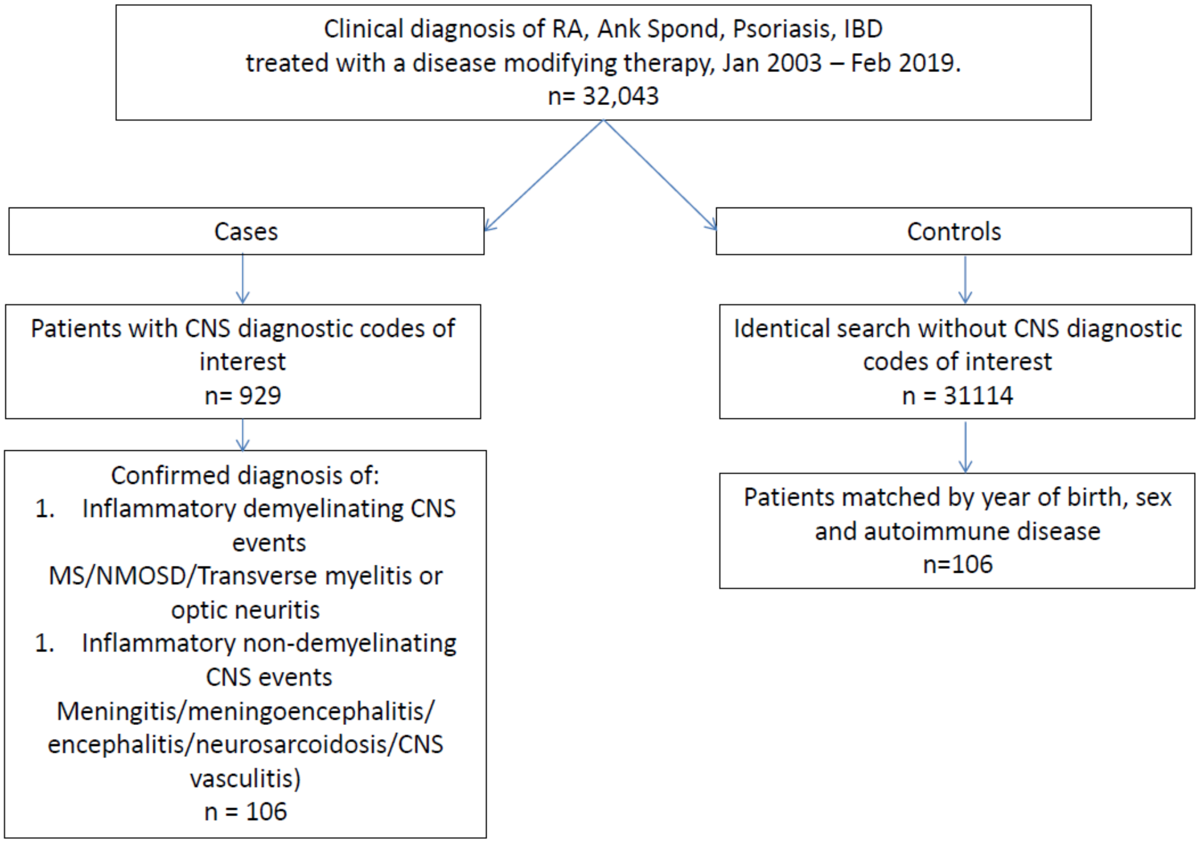


© 2020 Kunchok A et al. *JAMA Neurology.*

**eFigure 2: Study Design**

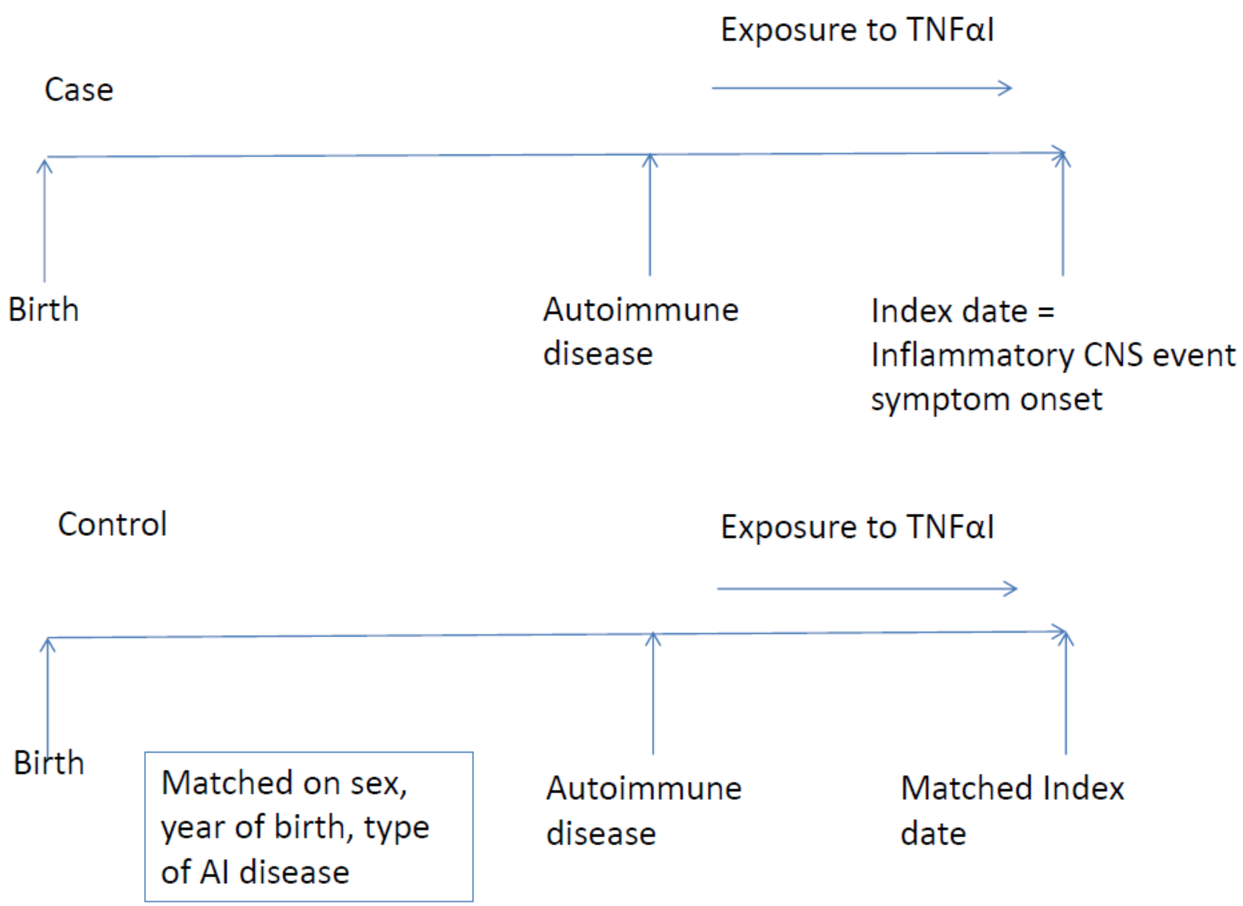


© 2020 Kunchok A et al. *JAMA Neurology.*

**eTable 1: Disease Modifying Therapies Use by the Study Population at Any Point in Their Disease Course**

| Disease modifying therapies: |
|---|
| Leflunomide |
| Azathioprine |
| Methotrexate |
| Hydroxychloroquine |
| Minocycline |
| Sulfasalazine |
| Gold |
| Cyclophosphamide |
| Mycophenolate |
| Cyclosporine |
| Anakinra |
| Sekukinumab |
| Ustekinumab |
| Abatacept |
| Rituximab |
| Tocilizumab |
| Natalizumab |
| Vedolizumab |
| Apremilast |
| Secukinumab |
| Ixekizumab |
| Brodalumab |
| Guselkumab |
| Tilrakizumab |
| Mesalamine |
| Etanercept |
| Infliximab |
| Adalimumab |
| Golimumab |
| Certolizumab |

© 2020 Kunchok A et al. *JAMA Neurology.*

**eTable 2: Exclusions From CNS Diagnostic Code Search for Cases**

| Reason for exclusion | n |
|---|---|
| **Cases not occurring during study period:** (prior to 2003 or prior to autoimmune disease onset) | 148 |
| **Malignancy:** Primary CNS malignancy (glioma, lymphoma, meningioma); paraneoplastic CNS disease; systemic malignancy (solid organ or haematological); secondary CNS (metastatic) disease | 22 |
| **Non-neurological diagnosis:** evaluation for MS negative, influenza, infectious mononucleosis, postural hypotension, spondylitis, chronic pain, systemic sarcoidosis but evaluation for CNS disease negative, systemic vasculitis but evaluation for CNS disease negative | 437 |
| **Other neurological disorder:** ALS; myasthenia gravis, cerebral palsy; CIDP; migraines; myositis; peripheral neuropathy; CNS ischaemia; leukodystrophy; leukoencephalopathy; epilepsy (non-autoimmune); PRES; pachymeningitis due to CSF leak or IgG4 disease; skull base osteomyelitis; trigeminal neuralgia; tremor; haemangioma; cavernoma; osmotic demyelination syndrome; depression; narcolepsy; dementia; PML; toxic-metabolic encephalopathy; arachnoiditis; compressive myelopathy; infectious transverse myelitis; meningitis secondary to drugs, bacterial or viral meningitis; surgical meningitis(post ENT or neurosurgical intervention); optic neuropathy secondary to other causes (drugs, ischaemia, glaucoma, non-arteritic ischaemic, sellar lesions, trauma, IgG4) | 155 |
| **Lacking ancillary confirmative tests:** lack of confirmatory CSF or MRI | 7 |
| **Autoimmune disease not confirmed:** autoimmune diagnosis removed or alternative autoimmune disease | 23 |
| **Immunodeficiency or immunocompromised:** solid organ transplant, stem cell transplant, congenital (common variable) or acquired immunodeficiency (HIV) | 28 |
| **Cases meeting inclusion criteria but consent for research refused** | 3 |
| **Total** | 823 |

© 2020 Kunchok A et al. *JAMA Neurology.*

**eTable 3: Cases Stratified by Inflammatory Demyelinating and Non-demyelinating CNS Events for Each Systemic Autoimmune Disease**

| Autoimmune disease | Total cases | Inflammatory demyelinating CNS events | Inflammatory non-demyelinating CNS events |
|---|---|---|---|
| | n | n (%) | n (%) |
| Rheumatoid arthritis | 48 | 26 (54) | 22 (45) |
| Ankylosing spondylitis | 4 | 1 (25) | 3 (75) |
| Psoriasis and Psoriatic arthritis | 21 | 12 (57) | 9 (43) |
| Crohn's disease | 27 | 14 (53) | 13 (48) |
| Ulcerative colitis | 6 | 3 (50) | 3 (50) |

© 2020 Kunchok A et al. *JAMA Neurology.*
